# Special Issue: “Organic Reactions in Green Solvents”

**DOI:** 10.3390/molecules21111527

**Published:** 2016-11-15

**Authors:** Jonathan Sperry, Joaquín García-Álvarez

**Affiliations:** 1 Centre for Green Chemical Science, School of Chemical Sciences, University of Auckland, 23 Symonds Street, Auckland 1000, New Zealand; 2 Laboratorio de Compuestos Organometálicos y Catálisis (Unidad Asociada al CSIC), Departamento de Química Orgánica e Inorgánica, (IUQOEM), Centro de Innovación en Química Avanzada (ORFEO-CINQA), Facultad de Química, Universidad de Oviedo, Oviedo E-33071, Spain

**Keywords:** bio-based solvents, water, deep eutectic solvents, ionic liquids, green organic synthesis

## Abstract

To overcome the well-established drawbacks of conventional organic solvents (toxicity, non-biodegradability, flammability, accumulation in the atmosphere) remarkable research efforts have been recently devoted to the replacement of traditional organic reaction media by the so-called *Green Solvents*. In this sense, the choice of a safe, non-toxic, biorenewable and cheap reaction media is a crucial goal in organic synthesis. Thus, this Special Issue on “*Organic Reactions in Green Solvents*” has been aimed to showcase a series of stimulating contributions from international experts within different sub-areas of organic synthesis in *Green Solvents* (ranging from metal- to organo-catalyzed organic reactions).

The development of new synthetic organic processes which are able to maximize the chemo- and regioselectivities, functional group tolerance and reaction yields has always represented a major goal in organic chemistry, either in academia or industry [1]. However, the last two decades have witnessed an increasing interest in the resolution of new challenges associated not only with productivity but also with the prevention and reduction of the ecological impact of chemical processes [2]. Thus, reactions should be now atom economical [3], catalytic [4], safe for both humans and the environment, and designed with energy efficiency (if possible, synthetic methods should be conducted at ambient pressure and temperature) [5]. Among these objectives, the *Green Chemistry Principles*, first postulated by Warner and Anastas [6], are driven *Chemistry* to reduce waste, re-use materials and increase sustainability. Besides undesired side products generated by stoichiometrically added reagents, chemical waste is mainly derived from solvents, which are necessary in the majority of the reactions to (*i*) accomplish homogeneity of substrates; (*ii*) prevent the formation of unwanted by-products through dilution; (*iii*) guarantee fast and safe conversions; and (*iv*) control the heat flow of the reaction [7]. For example, in the field of pharmacological organic synthesis, it has been estimated that up to 85% of the total involved mass is comprised of solvents [8]. Furthermore, in these synthetic processes, the recovery of the solvent usually ranges from 50 to 80% and therefore produces huge amounts of waste and consumes enormous quantities of petroleum-based chemicals [9]. Due to these low recovery rates, the depletion of petroleum sources and the toxic or ecologically damaging properties of most organic solvents, there has been a growing interest in changing to safer and more renewable *Green Solvents* [10,11,12].

This Special Issue includes five original research articles and one review article covering different topics ranging from metal- and organo-catalyzed organic reactions in *Green Solvents* to solvent-free green techniques. Firstly, the use of ionic solvents (traditional ionic liquids or *Deep Eutectic Solvents*) is discussed in three articles. These include the development of a greener synthesis of 2-aminoimidazoles using *Deep Eutectic Solvents* (*DESs*) as biorenewable and “innocent” reaction media. In this article Capriati, Salomone and co-workers have demonstrated that the use of choline chloride-based *DES* enables the direct isolation of highly-substituted 2-aminoimidazoles in good yields by employing a simple work-up procedure (filtration and crystallization) which allows for the *DES* to be recycled [13]. Liu et al. introduced a similar condensation approach for the synthesis of other dinitrogenated heterocycles [3,4-dihydropyrimidin-2(1*H*)-ones] employing, in this case, a Brønsted acid ionic liquid as catalyst under solvent-free conditions [14]. It is important to highlight that the ionic catalyst was recycled up to six consecutive cycles. Furthermore, Smiglak and co-workers have found that a biphasic system containing an ionic liquid (both imidazolium and pyridinium salts are employed) not miscible with the substrate (1-octene) is the reaction media of choice to accomplish the hydrosilylation of the desired olefin catalyzed by inorganic platinum or rhodium complexes [15]. The results reported in this article proved that this biphasic system allows the recycling of the metal catalyst, reducing the costs and decreasing the amount of catalyst needed per mole of product.

Secondly, the use of water as green solvent is also highlighted in this Special Issue by two different contributions. In this sense, Stavber et al. reported the efficient transformation of tertiary benzyl alcohols into the corresponding vicinal halo-substituted derivatives using *N*-halosuccinimides [16]. Interestingly, the efficiency of the reaction was increased when water was used as solvent, thus disclosing a new example of an accelerated organic reaction in aqueous media. In a review article, Santi and co-workers discuss the use of water as a natural and environmentally-friendly reaction media to perform organoselenium chemistry, putting the focus on the use of this medium to (*i*) accomplish the recyclability of the system; (*ii*) control the selectivity of the process; and (*iii*) enhance the reaction rate [17].

Finally, Eldeab et al. reported a solvent-free microwave irradiation method as a green technique for the synthesis of new thiopyridine arabinoses [18]. Moreover, the authors described the antimicrobial activity of all synthesized compounds against a panel of Gram-negative and Gram-positive bacterial strains.

In summary, the collection of original research and review articles included in this themed issue offer a broad view of the state-of-the-art in *Organic Reactions in Green Solvents*, highlighting the enormous scope for advancement and application in this field. To conclude, we would like to express our gratitude to all the authors for their enthusiastic response to this Special Issue and for their outstanding contributions. Last but not least, we would also like to acknowledge the excellent work of all the anonymous reviewers and the staff from the Editorial Office of *Molecules*, who were exceptionally helpful during the production of this Special Issue.
